# Syncope in a Dog for Five Hours as the Result of a Chloral and Morphine Mixture

**Published:** 1897-01

**Authors:** 


Syncope in a Dog for Five Hours as the Result of a Chloral and Morphine Mixture. A bulldog weighing 15 kilogrammes and six years old had a tumor on the right side of his nose about the size of a hickory-nut. As an anæsthetic, Mr. Pecus gave him a three-centigramme injection of morphine and administered to him an injection of 15 grains of chloral dissolved in the water of (flux) linseed. A half-hour later, as the animal was not completely insensible, he injected another centigramme of morphine. The operation was performed with the greatest facility and without any trouble due to anæsthesia, and the dog was placed on a bed in order to let him come to. Profound narcosis followed. The dog was restless, with irregular and spasmodic respiration, which stopped completely at times. After several hours the animal was revived by means of artificial respiration, drawing out the tongue and a subcutaneous injection of two milligrammes of pilocarpine and strychnine, mixed.—*Annales de Mét. Vét.*, March, 1896.

